# Neutrophil killing of *Mycobacterium abscessus* by intra- and extracellular mechanisms

**DOI:** 10.1371/journal.pone.0196120

**Published:** 2018-04-19

**Authors:** Kenneth C. Malcolm, Silvia M. Caceres, Kerstin Pohl, Katie R. Poch, Audrey Bernut, Laurent Kremer, Donna L. Bratton, Jean-Louis Herrmann, Jerry A. Nick

**Affiliations:** 1 Department of Medicine, National Jewish Health, Denver, CO, United States of America; 2 Department of Medicine, University of Colorado, Denver, Aurora, CO, United States of America; 3 Institut de Recherche en Infectiologie de Montpellier (IRIM), Centre National de la Recherche Scientifique UMR 9004, Université de Montpellier, Montpellier, France; 4 INSERM, IRIM, Montpellier, France; 5 Department of Pediatrics, National Jewish Health, Denver, CO, United States of America; 6 Infection et Inflammation Chronique (2I), Université de Versailles St Quentin, INSERM, Université Paris-Saclay, Versailles, France; Hospital for Sick Children, CANADA

## Abstract

*Mycobacterium abscessus*, a rapidly growing nontuberculous mycobacterium, are increasingly present in soft tissue infections and chronic lung diseases, including cystic fibrosis, and infections are characterized by growth in neutrophil-rich environments. *M*. *abscessus* is observed as two distinct smooth and rough morphotypes. The environmental smooth morphotype initiates infection and has a relatively limited ability to activate neutrophils. The rough morphotype has increased virulence and immunogenicity. However, the neutrophil response to the rough morphotype has not been explored. Killing of the smooth and rough strains, including cystic fibrosis clinical isolates, was equivalent. Neutrophil uptake of *M*. *abscessus* was similar between morphotypes. Mechanistically, both rough and smooth morphotypes enhanced neutrophil reactive oxygen species generation but inhibition of NADPH oxidase activity did not affect *M*. *abscessus* viability. However, inhibition of phagocytosis and extracellular traps reduced killing of the smooth morphotype with lesser effects against the rough morphotype. Neutrophils treated with *M*. *abscessus* released a heat-labile mycobactericidal activity against the rough morphotype, but the activity was heat-tolerant against the smooth morphotype. Overall, *M*. *abscessus* stimulates ineffective neutrophil reactive oxygen species generation, and key mechanisms differ in killing of the smooth (phagocytosis-dependent, extracellular traps, and heat-tolerant secreted factor) and rough (extracellular traps and a heat-labile secreted factor) morphotypes. These studies represent an essential advancement in understanding the host response to *M*. *abscessus*, and help explain the recalcitrance of infection.

## Introduction

*Mycobacterium abscessus* is a rapidly growing nontuberculous mycobacterium (NTM) whose incidence of infection has increased over the past decades, especially in patients with chronic lung disease [1–3]. This problem is of great concern due to the high intrinsic resistance of *M*. *abscessus* to most available antibiotics and to sanitizing solutions [4–7]. *M*. *abscessus* infections are characterized by growth in highly inflamed tissues [8], implicating the neutrophil as important in the host response. Supporting this conclusion, patients with cystic fibrosis (CF), a disease that is characterized by persistent neutrophil-mediated inflammation, are highly susceptible to *M*. *abscessus* [1–3,9].

NTM, including *M*. *abscessus*, can exist in two distinct morphotypes defined by their colony morphology on agar. The smooth morphotype is believed to be the environmental strain [10], but can convert to a rough morphotype as an adaptation to unknown growth conditions or selective pressures. This morphological change is reflected by the reduction or elimination of glycopeptidolipids (GPL) in the cell wall due to mutations or deletions in the genes responsible for GPL production or transport [11,12]. The *M*. *abscessus* smooth morphotype has greater biofilm forming potential, but is more susceptible to immune control by macrophages [13–15]. The rough morphotype has greater immunogenicity and virulence [13–17]. Macrophages infected with smooth *M*. *abscessus* produce little pro-inflammatory cytokines and control infection; in contrast, macrophages infected with rough *M*. *abscessus* produce copious pro-inflammatory cytokines and mycobacteria survive and grow intracellularly [13–15,17–20]. Furthermore, *in vivo* infection models confirm the virulence of rough, but not smooth *M*. *abscessus* [13,15,16,21,22] and persistent infection was associated with conversion to the rough morphotype in animal models [22–24] and in patients with lung infections [10,25–27]. We recently described increased virulence and granuloma formation of the rough morphotype in a zebrafish model of infection [21,28]. Despite the prevalence of neutrophils in *M*. *abscessus* infections, the neutrophil response to *M*. *abscessus* is poorly studied, and the basis of the virulent phenotype of the rough morphotype is poorly understood; however, in a zebrafish model neutrophils are important components of mycobacterial clearance [28].

Neutrophils induce a variety of anti-microbial responses following exposure to pathogens and pathogen-derived factors. Among these are generation of reactive oxygen species (ROS) and formation of neutrophil extracellular traps (NETs), which are composed of chromatin and the contents of neutrophil granule proteins [29], including enzymes responsible for infection control, such as elastase and myeloperoxidase. Upon stimulation, NETs are released into the extracellular space and are believed to immobilize pathogens for eventual immune cell clearance. In addition, neutrophils release antimicrobial peptides and enzymes.

The current study addresses the neutrophil bactericidal responses to the smooth and rough morphotypes of *M*. *abscessus* to help understand its virulence. Neutrophils killed the smooth and rough morphotype equally well, in contrast to reported killing by macrophages, in which only the smooth morphotype is killed [13–15]. Induction of ROS appears insufficient to kill either morphotype. However, NET formation is effective for the control of both *M*. *abscessus* morphotypes, while inhibiting uptake led to reduced killing of smooth *M*. *abscessus*. Furthermore, *M*. *abscessus* induced a neutrophil extracellular mycobactericidal activity. These studies indicate distinct and common neutrophil killing mechanisms of *M*. *abscessus* morphotypes, and suggest the potential for injurious release of neutrophil products during infection. Delineating the neutrophil responses to this emerging pathogen can lead to a better understanding of host-pathogen interaction and aid in formulating therapeutic strategies for *M*. *abscessus* control.

## Experimental procedures

### Bacterial strains, media and culture conditions

*M*. *abscessus* ssp. *abscessus* (ATCC strain 19977/ CIP104356T) was propagated from frozen aliquots in 7H9 broth supplemented with 0.5 g/l bovine albumin fraction V, 0.2 g/l dextrose,0.3 mg/l catalase (ADC; BD Biosciences), 2% glycerol and 0.05% Tween-80 at 33°C with shaking at 300 rpm for 3–5 days. The rough morphotype was isolated from ATCC 19977. GPL production profiles were confirmed for smooth and rough *M*. *abscessus* using thin-layer chromatography [15,30]. *M*. *abscessus* mCherry was produced as described [21]. Cultures were adjusted to OD_600_ of 1.0 in PBS containing Ca^2+^ and Mg^2+^, corresponding to approximately 1 x10^8^ cfu/ml. Clinical isolates were obtained from the Colorado Cystic Fibrosis Research and Development Program Culture, Biorepository, and Coordinating Core at National Jewish Health. Large aggregates were allowed to settle and only washed cultures containing single cells or small clumps were used; some assays were repeated using cultures sonicated using six one-second bursts of a Fisher Sonic Dismembrator 100 at approximately 4 W output as an alternative for obtaining single cells and smaller aggregates.

### Neutrophil isolation

Neutrophils were isolated from healthy volunteers by the plasma Percoll method as previously described [31], and were washed and resuspended in Krebs-Ringer phosphate-buffered saline with dextrose (154 mM NaCl, 5.6 mM KCl, 1.1 mM MgSO_4_, 2.2 mM CaCl_2_, 0.85 mM NaH_2_PO_4_, 2.15 mM Na_2_HPO_4_ and 0.2% dextrose). Cells were confirmed to be >98% pure by visual inspection of cytospins, and resulting gene arrays had negligible expression of genes specific to peripheral blood mononuclear cells. These studies were approved by the National Jewish Health Institutional Review Board, and written informed consent approved by the National Jewish Health Institutional Review Board was obtained from all neutrophil donors. The study was conducted in accordance with the Declaration of Helsinki.

### Killing assay

Neutrophils were suspended in RPMI supplemented with 10 mM HEPES, pH7.4, and 2% pooled heat-inactivated platelet-poor plasma (complete RPMI). Sample tubes contained cells (1x10^6^) suspended in complete RPMI and the respective bacteria at a multiplicity of infection (MOI) of approximately 1:1 in a 0.1 ml volume. Inhibitors were preincubated with neutrophils for 10 min prior to adding bacteria. Tubes were initially centrifuged at 4000 g for 1 min and pelleted cells incubated at 37°C for 5 min to promote bacteria-neutrophil interaction, followed by resuspension and incubation for up to 2 hr, as indicated. These experimental conditions promote synchronous, close contact of mycobacteria and neutrophils. Triton X-100 (0.1%) in 0.9% saline was added to inactivate neutrophils and aid in mycobacterial dispersion. Following vortex mixing and serial dilutions in saline, *M*. *abscessus* was plated on 7H10 agar supplemented with OADC and incubated at 37°C. Colonies were counted 3 to 5 days after plating, and compared to colony counts at initiation of infection.

### Cytokine and chemokine release

Secretion of cytokines and chemokines were determined from cell supernatants after 2h and 4h stimulation with *M*. *abscessus* at a MOI of 10:1. Products were quantified by ELISA for TNFα, IL8, IL1β, and CCL4/MIP1β (ELISATech).

### Superoxide release and intracellular reactive oxygen species (ROS) assays

Extracellular superoxide anion was assayed by cytochrome *c* reduction, as previously described [32,33]. Cells were stimulated with *M*. *abscessus* at a MOI of 10:1 for 15 and 60 min, or without *M*. *abscessus*. Intracellular ROS was measured using neutrophils loaded with 10 μM CM-H2DCFDA for 20 min; labeled cells were washed and resuspended in complete RPMI and 2 x 10^5^ cells were allowed to settle for 20 min in wells of a black 96-well plate at 37°C. Cells were incubated with DMSO, diphenylene iodonium (DPI; 10 μM), or cytochalasin D (5 μg/ml). Bacteria were added at MOI 10:1, centrifuged at 110 x g for 1 min and placed in a BioTek FL800 plate reader; reads were performed every 5 min for up to 2 hours at 37°C at excitation/emission of 485/528. Area-under-the-curve values were generated using GraphPad Prism 4.0.

### Phagocytosis of *M*. *abscessus*

For analyzing bacterial localization by microscopy, neutrophils were incubated with FITC-labeled *M*. *abscessus* (FITC-Mab) for 1 hour at an MOI of 5:1 [33]; cells were adhered to microscope slides by cytocentrifugation, and air-dried. FITC-Mab was prepared by incubating washed *M*. *abscessus* with 30 μg/ml FITC in 0.1 M sodium bicarbonate buffer, pH 9.6 for 30 min at room temperature in the dark. FITC-Mab were sonicated, as described above, washed two times, and resuspended in 0.6 ml saline or complete RPMI/ 0.02% Triton X-100 before dilution to OD_600_ = 1.6–1.7, and further diluted 1:1 in complete RPMI. Final Triton X-100 concentration in the experiments never exceeded 0.001%, which had no effect on cell viability (not shown). Slides were processed for elastase immunofluorescence, as described below. For flow cytometric analysis, neutrophils (1 x 10^6^) and FITC-Mab were mixed at an MOI of approximately 5:1. Samples were processed as described above in the killing assay with a brief centrifugation and incubation before resuspending the cells, and incubated over a period of 5 to 60 min. Reactions were stopped with an equal volume of ice-cold complete RPMI. Neutrophils were stained with eFluor 450-labeled anti-CD16 (CB16), and fixed in 1% formaldehyde/ 3% sucrose. To distinguish intracellular and extracellular staining, flow analysis was compared in duplicate samples in both the absence (total staining) and presence (internal staining) of 0.4% trypan blue; the difference of these is external staining. Analysis was performed on an LSRII flow cytometer (BD Biosciences) and FlowJo software (TreeStar). Neutrophils were gated to exclude cellular debris and planktonic and aggregated bacteria.

### LDH cytotoxicity assay

Neutrophil were pelleted at 20,000 x g at the indicated times and the supernatants retained for use in the LDH Cytotoxicity Assay Kit (Pierce). Results are depicted as % total LDH with a neutrophil detergent lysate used for the maximal LDH.

### Neutrophil extracellular DNA assays

DNA release from live cells was detected from neutrophils prepared and stimulated as described above, treated with DMSO, DPI (10 μM) or DNase (100 units/ml), and incubated with each bacteria at an MOI of 5 at 37°C with rotation. After 2h 2 x 10^5^ cells were removed to a glass-bottomed slide (ibidi), and incubated for 30 min at 37°C in a humidified chamber; cells were incubated with 0.2 μM Sytox Orange or Sytox Green cell-impairment DNA-binding dyes, and cells were imaged at room temperature using a fluorescence microscope [34]. In some cases, FITC-labeled *M*. *abscessus* was used. Slides were imaged and analyzed for number of positive cells and total area of fluorescence using ImageJ. Extracellular DNA associated with neutrophil extracellular trap (NET) formation was detected using a modification of the method of Fuchs *et al*. [35], as previously described [33,34]. Briefly, purified human neutrophils (2.4 x 10^6^) in complete RPMI were treated with DMSO, DPI (10 μM), DNase (100 units/ml), or cytochalasin D (5 μg/ml) and incubated with each bacteria at an MOI of 5 at 37°C with rotation. At times from 1–4 hours limited nuclease digestion was performed with micrococcal nuclease (0.5 units/ml) for 10 minutes at 37°C. Nuclease activity was then stopped with 5 mM EDTA, and cellular debris was removed by centrifugation at 200xg for 10 min. Soluble DNA content was measured with the Quant-iT™ Picogreen assay (Invitrogen) or with Sytox Green. To identify NET constituents, cells were prepared and stimulated for 2 h as indicated above, and 2 x 10^5^ neutrophils were spotted on a glass microscope chamber slide (Ibidi) for 30 min at 37°C in a humidified chamber, stained with 0.2 μM Sytox Orange the final 10 min, and fixed in 2% formaldehyde for 10 min at room temperature. Cells were permeabilized in 0.2% Triton X-100, stained overnight with anti-elastase (abcam; ab-21595; 1:100), and visualized by confocal fluorescent microscopy after binding of Alexafluor 488-conjugated goat anti-rabbit antibody.

### Preparation of neutrophil conditioned media and the effect on *M*. *abscessus* viability

Neutrophils (10 x 10^6^) were suspended in 1 ml complete RPMI and treated with *M*. *abscessus* (1:1 MOI), peptidoglycan (10 μg/ml; Aldrich), or left untreated for 1.5 hours at 37°C. Cell suspensions were centrifuged at 300 x g for 10 min, 4°C; the supernatants were centrifuged again at 20,000 x g for 10 min, 4°C, passed through a 0.2 micron syringe filter, and stored at -20°C. These experimental conditions minimalize the potential effect of NETs, and do not cause excessive cell lysis, as determined by LDH release. The clarified supernatants were thawed at room temperature, and 100 μl samples removed. One sample was left at room temperature and another pairwise sample was incubated at 95°C for 10 min. *M*. *abscessus* (1 x 10^6^ cfu) was added to each room temperature conditioned medium sample, and incubated for 1 h at 37°C with occasional mixing. The reaction was diluted with 0.1% Triton X-100, and serial dilutions were plated. The cfus after exposure of *M*. *abscessus* to media from unstimulated and stimulated neutrophils were used to determine the percent killing.

### Statistical analysis

Data is presented as mean + SEM and analyzed by t-test, one-way ANOVA, or two-way ANOVA with Bonferroni post-tests, as noted in the Figure legends. Significance was set at a P-value of 0.05.

## Results

### Neutrophil killing of *M*. *abscessus*

**Fig 1:**
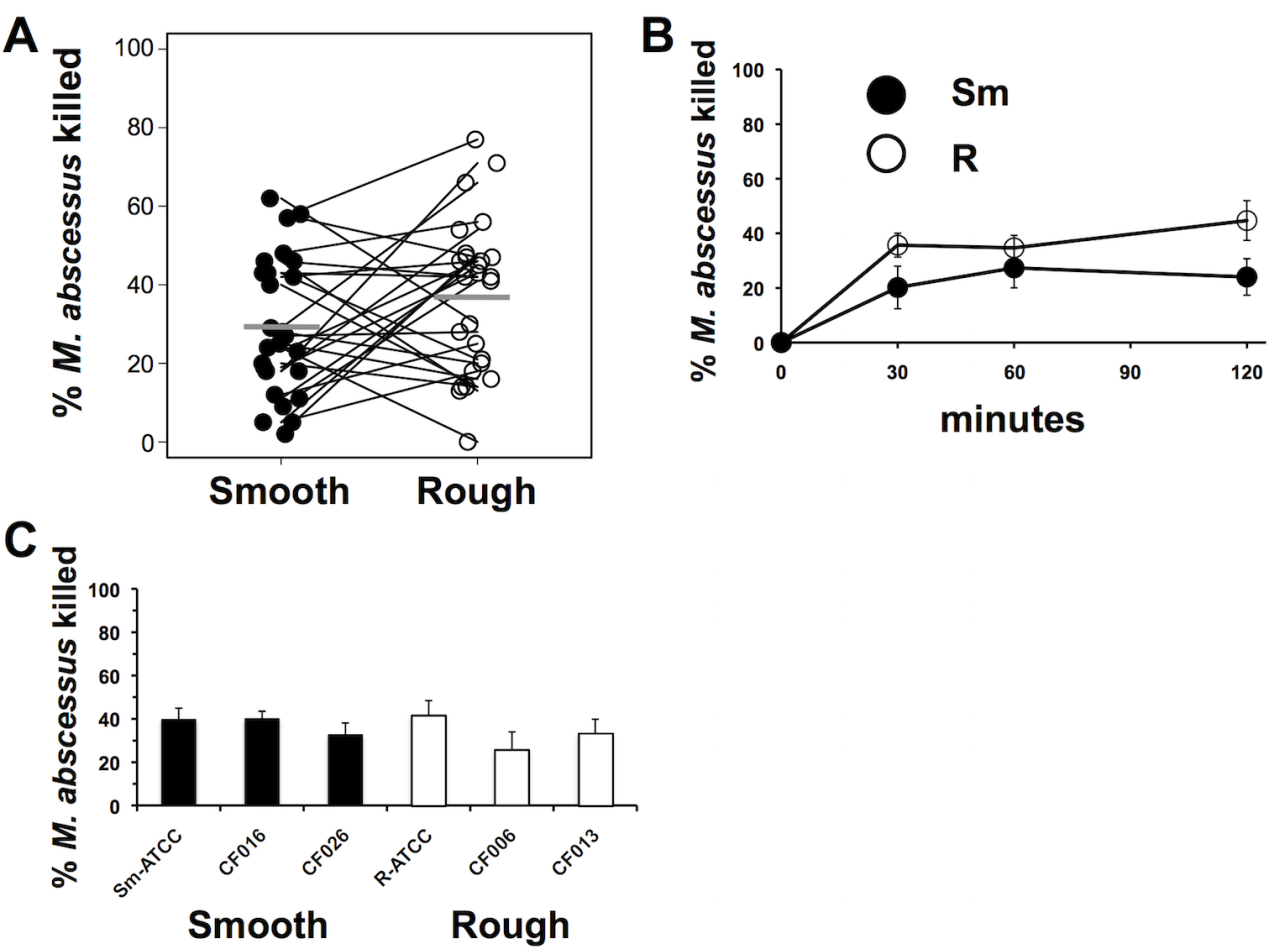
Neutrophil killing of *M*. *abscessus*. **(A)** Neutrophils were incubated with smooth (*closed circles*) and rough (*open circles*) *M*. *abscessus* for 1h, and surviving mycobacteria were compared to the inoculum. Connecting lines represent results from the same donor neutrophils; n = 26. Mean values are depicted by the gray bars. **(B)** Time course of killing of *M*. *abscessus* morphotypes by neutrophils; n = 6. **(C)** *M*. *abscessus* clinical isolates were assayed for killing by neutrophils for 1 h; smooth morphotypes (*closed bars*); rough morphotypes (*open bars*); n = 4–10. None of the differences were significantly different for the Fig 1 data by paired t-test.

Our previous work indicated that neutrophils poorly kill smooth *M*. *abscessus* compared to *S*. *aureus* [33]. However, little is known of the ability of neutrophils to control the rough morphotype. Rough and smooth morphotypes of *M*. *abscessus* were killed by neutrophils to a similar extent (**Fig 1A**). Both morphotypes were killed rapidly, with maximal killing by neutrophils occurring within 30 min to 1 hour of contact (**Fig 1B**), after which a plateau of killing was observed. We tested the possibility that susceptibility to neutrophil killing may also be dependent on virulence characteristics of the pathogen. Smooth and rough isolates derived from CF patients were similarly susceptible to neutrophil killing (**Fig 1C**). Killing represents the sum of a variety of neutrophil antimicrobial mechanisms; to gain a better understanding of *M*. *abscessus* immunity, we compared neutrophil antimicrobial functions between morphotypes.

## Phagocytosis of *M*. *abscessus* morphotypes

**Fig 2:**
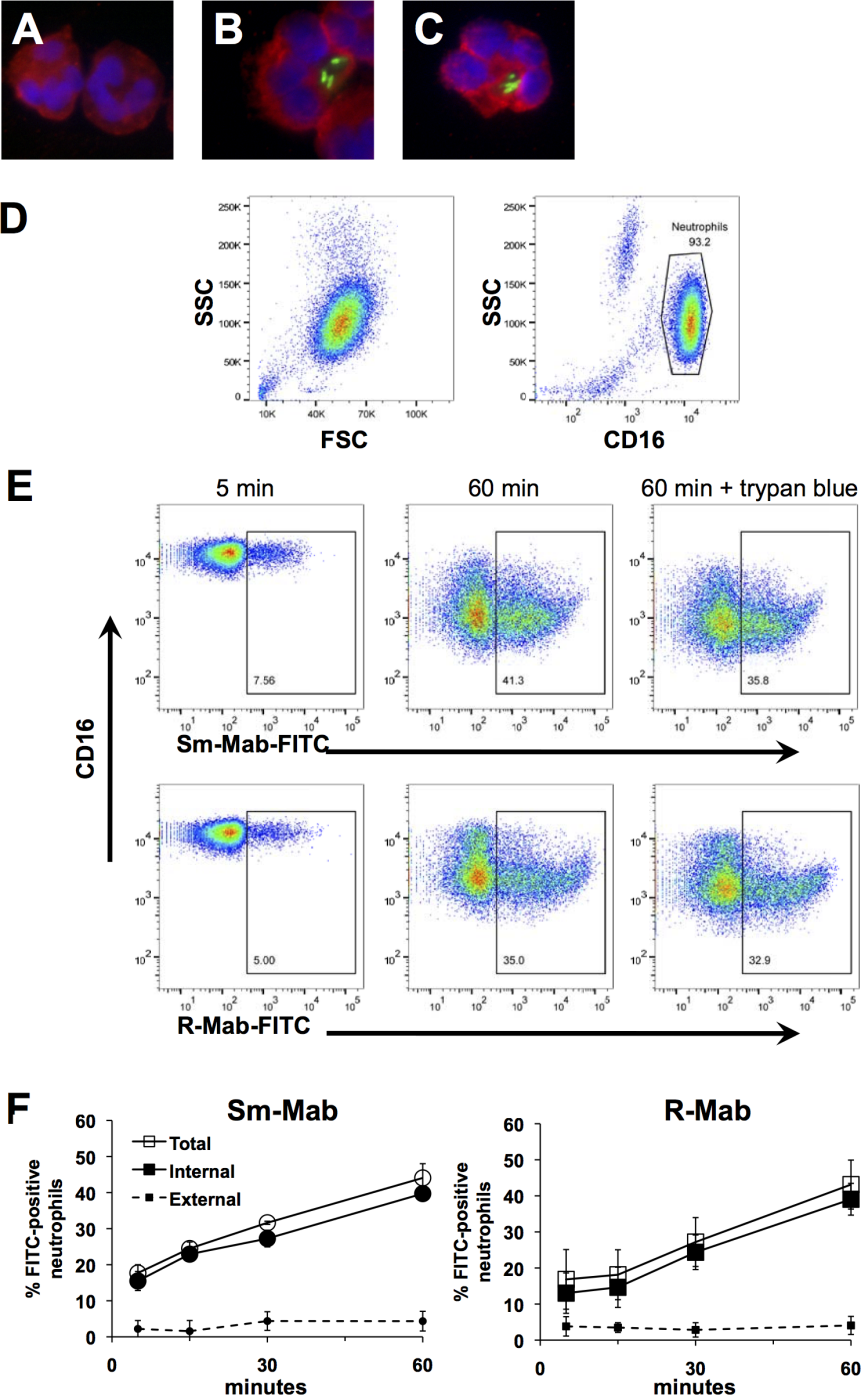
Phagocytosis of *M*. *abscessus* by neutrophils. **(A-C)** FITC-*M*. *abscessus* (*green*) was incubated with neutrophils for 1 h, and cells were cytocentrifuged on to glass slides, stained for elastase (*red*), and visualized by confocal microcopy. **(A)** Non-stimulated neutrophils, or after incubation with **(B)** smooth, and **(C)** rough FITC-*M*. *abscessus*. **(D)** Representative flow cytometry plots of isolated non-stimulated neutrophils showing forward scatter (FSC) and side-scatter (SSC) of non-stimulated neutrophils, and AlexaFluor-450-conjugated CD16. **(E)** Flow cytometric analysis of FITC-Mab association with neutrophils at 5 and 60 min; trypan blue was included to quench extracellular fluorescence. Examples of smooth (Sm-Mab; upper panels) and rough (R-Mab; lower panels) *M*. *abscessus* uptake are shown. **(F)** Time-dependent uptake of FITC-Mab. Neutrophils were incubated for the indicated time with smooth FITC-Mab (*left*) and rough FITC-Mab (*right*). Data represents the percentage of neutrophils positive for FITC for 3 independent experiments.

Fluorescence microscopy revealed phagocytosis of both smooth and rough *M*. *abscessus* by neutrophils (**Fig 2A–2C**). Human neutrophil elastase stained in non-nuclear areas surrounding *M*. *abscessus*, suggestive of phagosome formation. In contrast, non-infected neutrophils demonstrated more uniform elastase staining.

To quantify phagocytosis of *M*. *abscessus*, neutrophils were exposed to FITC-labeled *M*. *abscessus* and neutrophil-associated bacteria were measured by flow cytometry. Neutrophils were gated on CD16 and side scatter to exclude contaminating cells and free bacteria (**Fig 2D**). Examples of detection of neutrophil-associated *M*. *abscessus* are demonstrated at 5 min and 60 min (**Fig 2E**). Neutrophils associated with both morphotypes with similar kinetics and extent (**Fig 2F**). Trypan blue was used to distinguish between internal and external fluorescence. The measureable uptake at 5 min and the low level of quenching by trypan blue suggests efficient uptake of *M*. *abscessus*. Similar data was obtained using mCherry-labeled *M*. *abscessus* morphotypes that showed maximal uptake by 60 min (preliminary data). Furthermore, incubation with cytochalasin D, an inhibitor of phagocytosis, reduced the association of *M*. *abscessus* with neutrophils to less than 5% (unpublished data).

### Cell death occurs in response to rough *M*. *abscessus*

**Fig 3:**
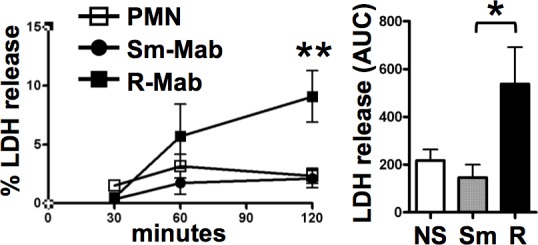
Rough *M*. *abscessus* is cytotoxic. Neutrophils were incubated with *M*. *abscessus* morphotypes for the indicated times (*left panel*). Samples were analyzed for LDH release, and compared to the total LDH from an equivalent amount of neutrophils. PMN, unstimulated neutrophils; Sm-Mab (*circles*), smooth; and R-Mab (*squares*), rough *M*. *abscessus*; **p<0.01 by two-way ANOVA and Bonferroni post-test comparing R-Mab to Sm-Mab and neutrophils alone at 120 min; n = 7. *Right panel*, AUCs were computed from data in the *left panel*; *p<0.05 by one-way ANOVA and Bonferroni post-test comparing R-Mab to Sm-Mab; NS, non-stimulated neutrophils.

Bacterial infection may cause neutrophil cell death. Incubation of neutrophils with smooth *M*. *abscessus* resulted in minimal necrotic cell death over 2 hours, while the rough morphotype induced a time-dependent increase in LDH release (**Fig 3**). These data are consistent with low cytotoxicity by smooth *M*. *abscessus* over 4 hours [33]. Therefore, late necrotic cell death is activated selectively by the rough morphotype.

### Cytokine response is similarly activated by both morphotypes

Other studies have observed a robust cytokine response of macrophages to the rough morphotype, while the smooth morphotype generated a weak response [18–20,36]. We compared the release of inflammatory cytokines (IL-1ß and TNFɑ) and chemokines (IL8 and CCL4/MIP1ß) by the two morphotypes as a measure of neutrophil activation. Surprisingly, no differences in neutrophil cytokine and chemokine release were observed (**S1 Fig**)[18–20,36]

### *M*. *abscessus* induces neutrophil ROS

**Fig 4:**
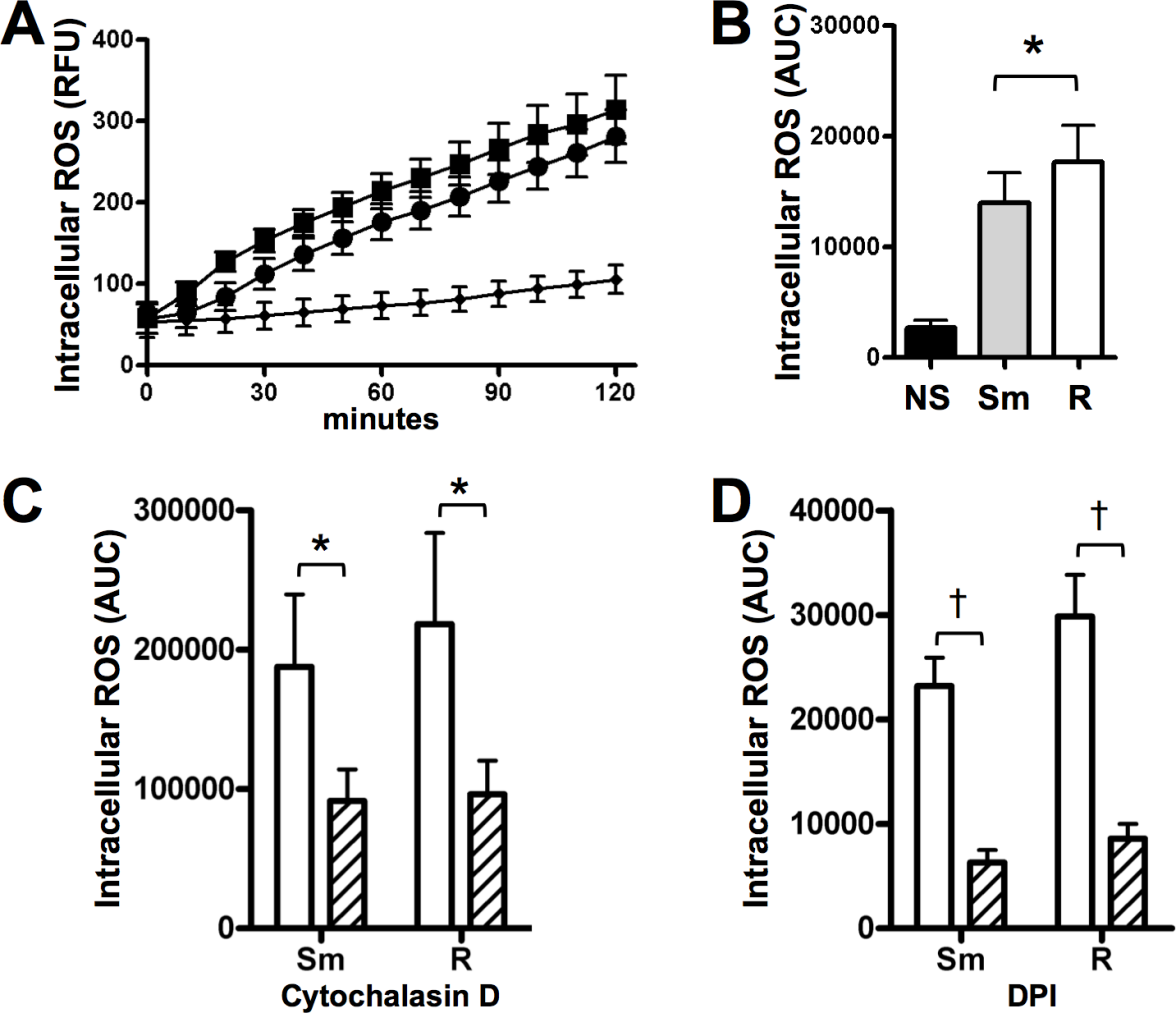
Reactive oxygen species (ROS) generated by neutrophils exposed to *M*. *abscessus*. **(A)** Intracellular ROS production is enhanced in the presence of smooth (*circles*) and rough (*squares*) *M*. *abscessus*; *small diamonds*, non-stimulated neutrophils; n = 8. **(B)** Areas-under-the-curve (AUC) were calculated for each condition in **A**; data analyzed by t-test; *p<0.05. Areas-under-the-curve were calculated for intracellular ROS curves of *M*. *abscessus*-infected neutrophils treated with **C)** cytochalasin D (*hatched*), and **D)** DPI (*hatched*); data analyzed by t-test; n = 3–9; *p<0.05, †p<0.001.

Neutrophil ROS generation is an important mechanism of pathogen killing. Because neutrophils were able to internalize *M*. *abscessus*, we measured intracellular ROS production. While both morphotypes led to intracellular ROS, rough *M*. *abscessus*-induced ROS production was more rapid and slightly more robust (**Fig 4A**). Taking the area-under-the-curve as an indication of the total response, rough *M*. *abscessus* produced significantly more intracellular ROS (**Fig 4B**). Extracellular superoxide generation was also measured in the presence of smooth and rough *M*. *abscessus*, and both *M*. *abscessus* morphotypes elicited similar superoxide release as *S*. *aureus* and PMA at one hour post-stimulation (not shown).

In order to determine the role of phagocytosis on intracellular ROS generation, we used cytochalasin D to block actin-dependent processes. Cytochalasin D reduced neutrophil intracellular ROS production by *M*. *abscessus* morphotypes (**Fig 4C**), as did DPI, an inhibitor of oxidant production (**Fig 4D**). Neither inhibitor affected naïve neutrophils over the same time course (not shown).

### *M*. *abscessus* induces neutrophil extracellular trap formation

**Fig 5:**
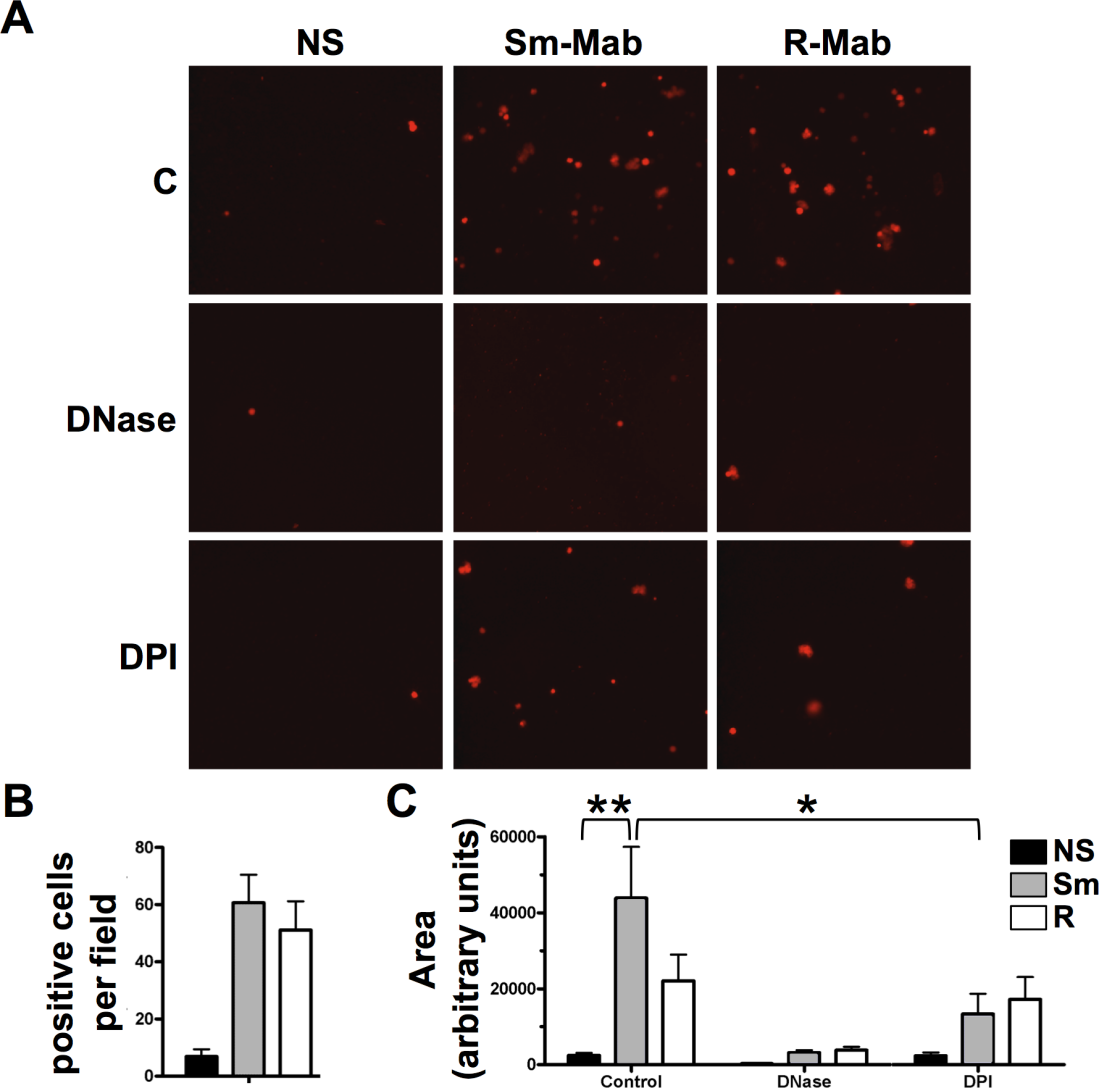
Release of DNA from live neutrophils by *M*. *abscessus*. **(A)** Neutrophils were left non-stimulated (NS) or exposed to smooth (Sm-Mab) and rough (R-Mab) *M*. *abscessus* for 2h in suspension, and plated on individual wells of a glass-bottom slide for 30 min. Cells were treated with DNase and DPI throughout infection, as indicated. Cell-impermeable Sytox Orange was added to each well, and fluorescence was monitored by microscopy. **(B)** The number of cells staining positive for Sytox Orange. **(C)** The area of positive fluorescence per field with three fields analyzed per experiment. Data analyzed by two-way ANOVA and Tukey’s multiple comparison test; n = 4; *p<0.05, **p<0.01.

**Fig 6:**
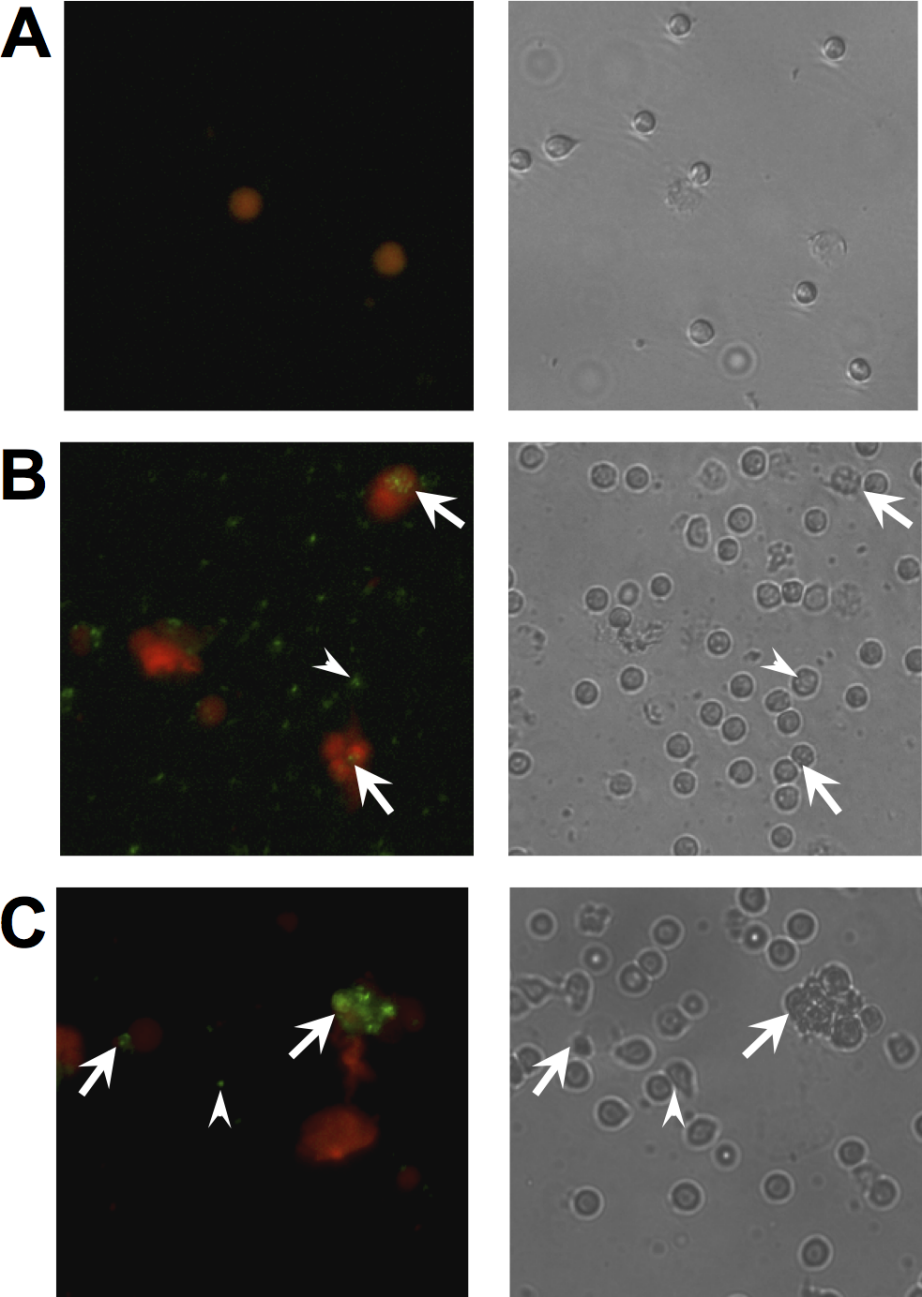
Localization of *M*. *abscessus* with neutrophils and extracellular DNA. **(A)** Untreated neutrophils; **(B)** smooth *M*. *abscessus*-treated neutrophils; and **(C)** rough *M*. *abscessus*-treated neutrophils. *Left panels*, Fluorescence imaging of neutrophils treated for 3 hours with FITC-labeled *M*. *abscessus* (*green*) and extracellular DNA stained with Sytox Orange (*red*), as in Fig 5A. *Right panels*, Phase contrast pictographs of same field. *Arrowheads*, location of FITC-labeled *M*. *abscessus* with apparent intracellular association; *arrows*, association of FITC-labeled *M*. *abscessus* with extracellular DNA. Data is representative of four independent experiments.

NETs are extracellular structures consisting of chromatin and neutrophil granule proteins, including elastase, and are involved in control of infections [29]. As in initial measure of NET formation, we observed DNA release from neutrophils in the presence of *M*. *abscessus* using a cell-impermeable DNA stain. Both morphotypes caused release of DNA (**Fig 5A**), and a similar number of cells stained positively for extracellular DNA (**Fig 5B**). When the area of released DNA was analyzed, the smooth morphotype stimulated significantly greater staining of extracellular DNA compared to non-stimulated neutrophils (**Fig 5C**); the area of released DNA by the rough morphotype was also consistently greater than control, but this trend was not significant (p = 0.054). The discrepancy between the area and number of cells with released DNA in rough-exposed neutrophils may be related to enhanced necrosis (Fig 2). Released DNA co-localized with *M*. *abscessus* (**Fig 6**, arrows), suggesting trapping of *M*. *abscessus*. Additionally, intact neutrophils were observed to associate with *M*. *abscessus* in the absence of extracellular DNA structures (**Fig 6**, arrowheads), which could represent phagocytosis or membrane association, as seen in Fig 2.

**Fig 7:**
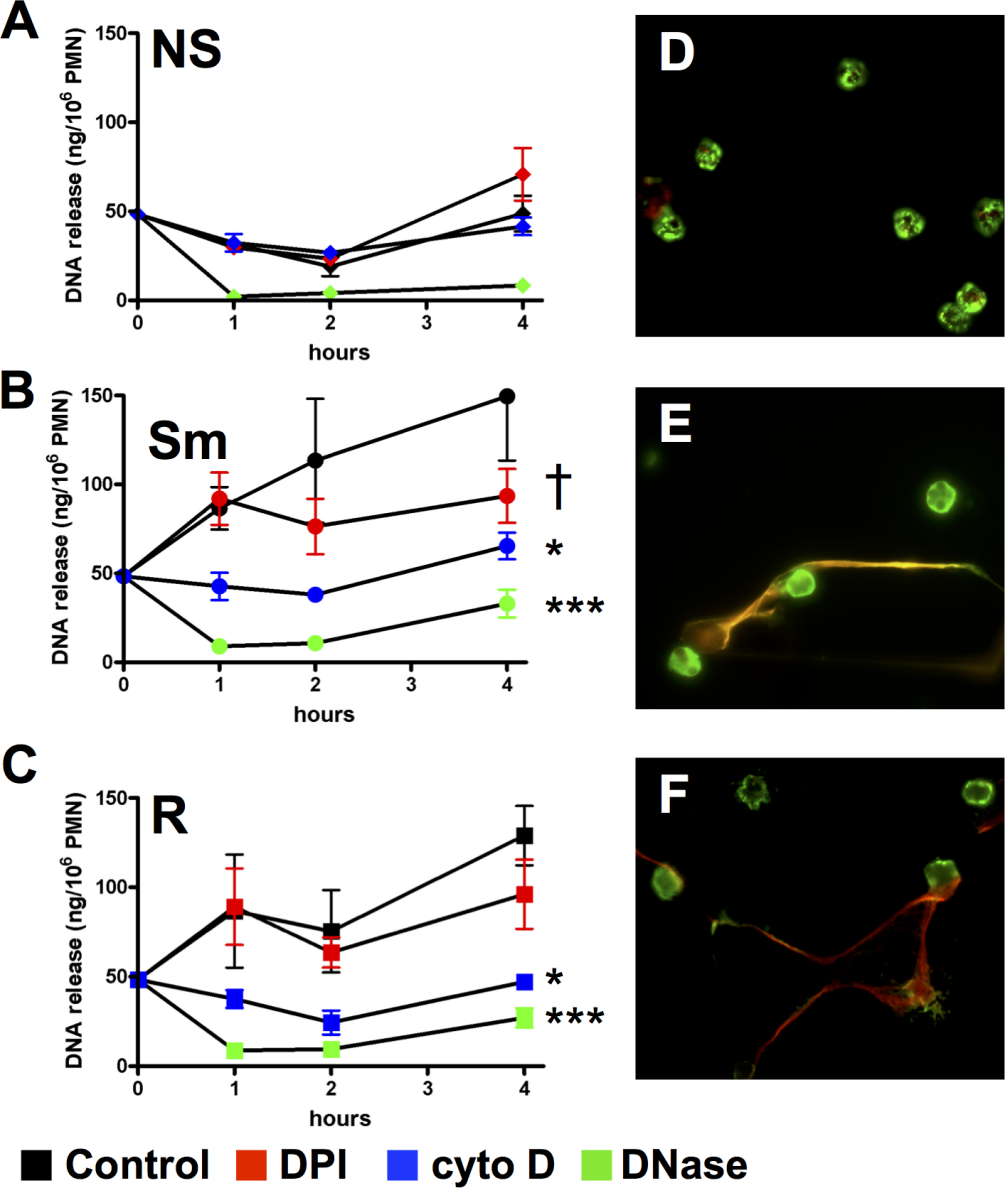
Extracellular DNA release induced by *M*. *abscessus*. Micrococcal nuclease-releasable extracellular DNA was quantified in **(A)** non-stimulated neutrophils (NS) and in neutrophils stimulated with **(B)** smooth (Sm) and **(C)** rough (R) *M*. *abscessus* for the indicted times. Neutrophils were pre-incubated with DPI (*red*), cytochalasin D (*blue)*, *and* DNase (*green*), as indicated. Data was analyzed by two-way ANOVA and Bonferroni post test versus the control (untreated) condition; n = 5; *p<0.05, and ***p<0.001 for all time points; †p<0.001 at 4h only. **(D-F**) NET formation by *M*. *abscessus* morphotypes. Neutrophils were left non-stimulated (**D**) or exposed to smooth (**E**) and rough (**F**) *M*. *abscessus* in suspension for 1h followed by plating on glass slides for an additional 2h. Sytox Red was added for 10 min, and cells were fixed and permeabilized. Slides were immunostained for elastase (*green*) and visualized under a 40X objective. Colocalized staining of extracellular elastase and extracellular DNA is observed in yellow.

DNase eliminated detection of NETs while inhibition of oxidant production with DPI only partially reduced NET release from neutrophils (**Fig 5A and 5C**). DPI-resistant NET formation has been reported after early exposure to *S*. *aureus* [37]. To verify microscopic findings, we measured the time-dependent release of DNA and no difference between morphotypes was observed (**Fig 7A–7C**). DPI had no effect on early NET formation in response to either morphotype. However, DNA release at 4 hours was partially inhibited by DPI in neutrophils stimulated only with smooth *M*. *abscessus*; DPI did not alter DNA release in response to the rough morphotype at any time point. These data are consistent with data shown in Fig 5, which was measured at 2.5 h. DNA release was eliminated in the presence of DNase and cytochalasin D at all times measured, suggesting that internalization is involved in this process (**Fig 7A–7C**). These extracellular DNA structures were confirmed as NETs using immunofluorescence microscopy. Co-localization of elastase and DNA in extracellular structures is consistent with NETs (**Fig 7D–7F**). These data suggest that NET release requires phagocytosis and is independent of ROS production at early time points.

### *M*. *abscessus* killing is blocked when disrupting NETs

**Fig 8:**
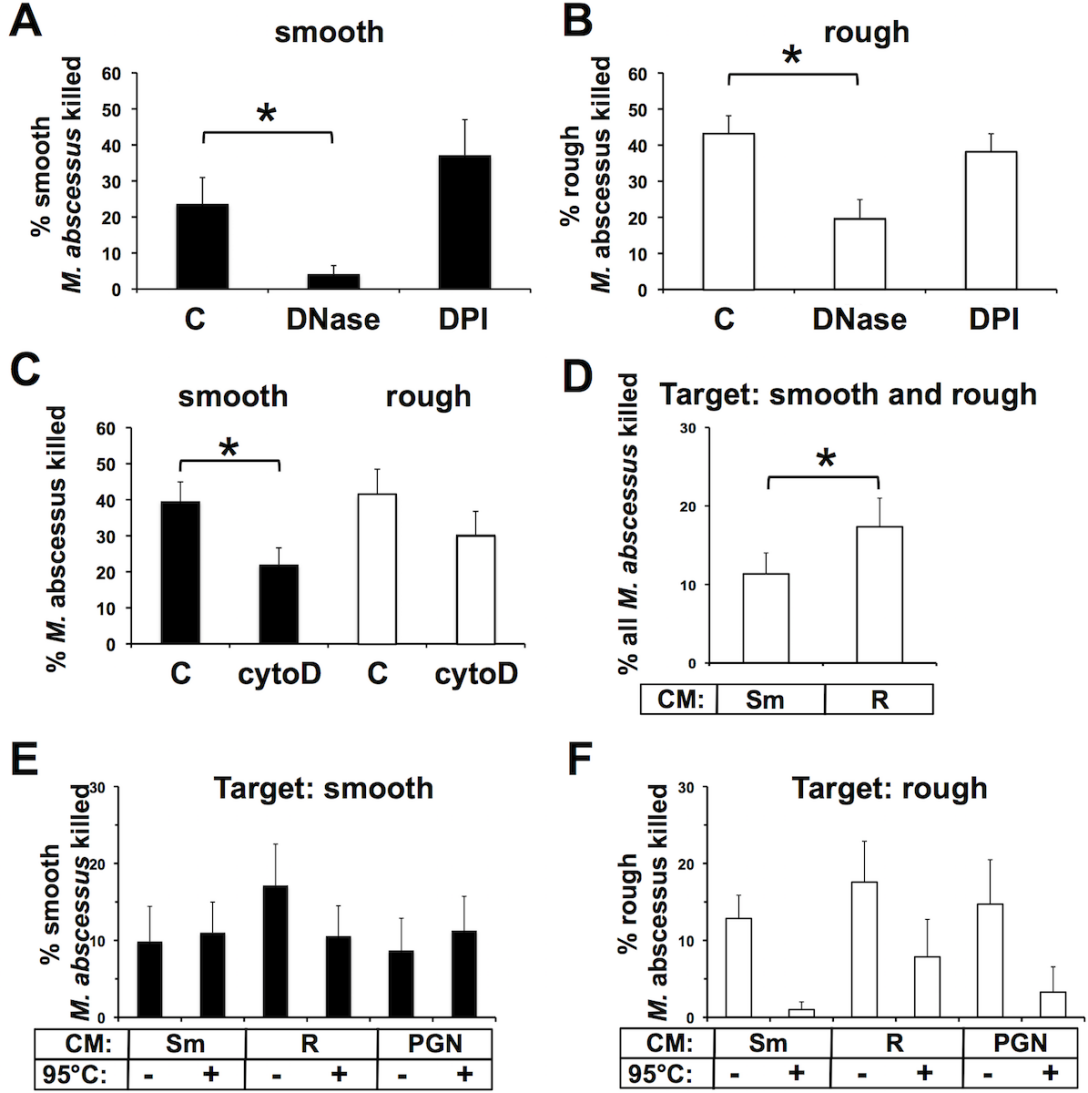
Intra- and extracellular neutrophil killing of *M*. *abscessus*. Neutrophils (n = 7) were pre-incubated with DNase (100 units/ml) or DPI (10 μM), or left untreated (C). Neutrophils were exposed to **(A)** smooth (n = 6) or **(B)** rough *M*. *abscessus* (n = 7) for 1h, and surviving mycobacteria were compared to the initial infection. **(C)** Neutrophils (n = 7) were treated with cytochalasin D (cytoD; 5 μg/ml) or left untreated (C) and killing of smooth (*closed bars*) and rough (*open bars*) *M*. *abscessus* was assessed after 1h. Panels A-C were analyzed by t-test; *P<0.05. **(D)** Targeting of *M*. *abscessus*, without regard for morphotype, by cell-free conditioned media (CM) from neutrophils treated with smooth *M*. *abscessus* (Sm) or rough *M*. *abscessus* (R), and represents composite data from **E** and **F**. Data analysis by paired t-test; n = 14. Morphotype-specific targeting of **(E)** smooth or **(F)** rough *M*. *abscessus* by conditioned media (CM) from neutrophils treated with smooth *M*. *abscessus* (Sm), rough *M*. *abscessus* (R), or peptidoglycan (PGN); conditioned media were incubated at 95°C, as indicated, before killing assays. Killing of each morphotype was normalized to that of *M*. *abscessus* exposed to supernatants from untreated neutrophils. Data were analyzed by two-way ANOVA; n = 7; an interaction (P = 0.01) was found for heat-treatment of CM for targeting rough *M*. *abscessus* (F), but not smooth (E).

To address the mechanism of killing of *M*. *abscessus*, inhibitors of NET formation, neutrophil ROS production, and phagocytosis were used. DPI, an inhibitor of ROS production, had no effect on the ability of neutrophils to kill *M*. *abscessus* (**Fig 8A and 8B**). DNase, which reduces the presence of NETs, inhibited killing of both morphotype (**Fig 8A and 8B**). However, a dichotomous effect was observed on morphotype killing in the presence of cytochalasin D, an inhibitor of phagocytosis; a trend in reduced killing of rough *M*. *abscessus* was seen (P = 0.07), while cytochalasin D partially and significantly inhibited killing of smooth *M*. *abscessus* (**Fig 8C**). These data suggest that smooth *M*. *abscessus* killing occurs via NETs, and partially via internalization, while rough *M*. *abscessus* is sensitive primarily to NETs.

### Conditioned medium from *M*. *abscessus* has mycobactericidal activity

The incomplete killing of *M*. *abscessus* in cytochalasin D-treated neutrophil suggests that neutrophils exhibit intracellular and extracellular killing activities. NETs appear to account for some of the extracellular bactericidal activity. To assess for additional extracellular bactericidal factors we treated neutrophils with *M*. *abscessus* and tested this conditioned media for mycobactericidal activity. The conditioned media isolated from neutrophils treated with both *M*. *abscessus* morphotypes killed target *M*. *abscessus* (**Fig 8D–8F**). The rough morphotype neutrophil medium showed a trend toward more effective killing of both individual morphotypes (**Fig 8E and 8F**). We then analyzed how conditioned media affected *M*. *abscessus* viability without regard for morphotype, and the aggregate killing was significantly greater with the rough *M*. *abscessus*-conditioned medium (**Fig 8D**). Media from neutrophils treated with peptidoglycan, a pathogen-associated molecule from Gram-positive *S*. *aureus*, also displayed bactericidal activity that was not different between morphotypes. To further characterize this extracellular factor, we incubated conditioned media at 95°C before performing mycobacterial killing assays. Heat-treatment of conditioned media from *M*. *abscessus*- or PGN-treated neutrophils did not affect its mycobactericidal activity against smooth *M*. *abscessus* (**Fig 8E**); however, heat-treatment of these conditioned media inhibited mycobactericidal activity against rough *M*. *abscessus* (**Fig 8F;** two-way ANOVA interaction for heat-treatment of CM, P = 0.01), particularly by neutrophil media conditioned by treatment with the smooth morphotype. These data suggest that both morphotypes induce distinct neutrophil-derived bactericidal activities that target either the smooth (heat-tolerant activity) or rough (heat-labile) morphotypes.

## Discussion

*M*. *abscessus* infections are distinguished by neutrophil-rich environments found in soft tissue abscesses and the CF lung. However, little is known about the neutrophil response to *M*. *abscessus*. Our previous work suggests that neutrophils promote smooth *M*. *abscessus* infections by limited clearance and through neutrophil-enhanced biofilm formation [33]. Through unknown mechanisms the smooth morphotype converts to a rough morphotype, and clinical and experimental data support a role for the rough morphotype in virulence [10,13–17,25–27]. Current data suggest that rough morphotypes, which lack GPL, have unique cell wall properties that enhance immunogenicity and virulence. However, the true role of each morphotype in immune response, disease progression, and antibiotic responses is poorly understood. Furthermore, the role of neutrophils, a cell central to the host response to *M*. *abscessus*, in the control of *M*. *abscessus* infection is largely unknown.

We have addressed some of these concerns in the present study. We have found that both intracellular and extracellular mechanisms contribute to neutrophil clearance of *M*. *abscessus*. NET formation appears to be the major bactericidal activity against *M*. *abscessus*, as DNase treatment during infection reduced bactericidal activity. Interestingly, NET formation proceeded when ROS was inhibited by DPI. Rapid ROS-independent NET release by *M*. *abscessus* is reminiscent of *S*. *aureus*, another abscess-forming pathogen common in CF [37], and of *Candida albicans* [38]. However, late NET formation was sensitive to DPI during smooth, but not rough, *M*. *abscessus* infection, although enhanced cytotoxicity may complicate detection of late extracellular DNA by rough *M*. *abscessus*. NET formation was also blocked by cytochalasin D, suggesting that internalization of *M*. *abscessus* is required for this process, which was previously reported for NET formation in response to opsonized *S*. *aureus* [39]. Importantly, the effects on killing by inhibitors of ROS, phagocytosis, and NET formation are mechanistically consistent in NET formation during *M*. *abscessus* infection; inhibition of phagocytosis by cytochalasin D inhibits NETs and interferes with killing, but DPI has no effect on killing or early NETs formation. The in vivo role of NETs in the control of *M*. *abscessus* infection needs to be further evaluated. In this regard, the zebrafish model of *M*. *abscessus* infection may be useful to directly observe the development of NETs during infection and to address their contribution in the physiopathology of the infection [21]. Likewise, ineffective, but robust, ROS production may contribute to tissue injury during *M*. *abscessus* infection.

Neutrophils are equally effective at killing the rough and smooth morphotypes. However, mechanistic differences in neutrophil killing of the rough and smooth morphotypes were observed. Specifically, inhibiting phagocytosis does not affect killing of the rough morphotype, whereas killing of the smooth morphotype was at least partially blocked. These data indicate that extracellular killing mechanisms targeting the rough morphotype are important. NET formation appears important in killing of both morphotypes, but the detection of extracellular mycobactericidal activities with different properties suggests that specific activities target each morphotype. Jena et al. described the sensitivity of mycobacteria to lysozyme and elastase, but with little added activity provided by β-defensins [40], and *M*. *abscessus* is resistant to the antimicrobial peptide LL37 [30]. However, we were not able to detect substantial *M*. *abscessus* killing by purified lysozyme, elastase, or their combination (not shown). Therefore, the identity of secreted mycobactericidal factors remains unknown. Our data does suggest that the presence of GPL on the cell wall of smooth *M*. *abscessus* may be one mechanism of resistance to clearance by this extracellular factor(s), as smooth *M*. *abscessus* is less sensitive to killing.

The ability of cytochalasin D to partially inhibit killing of smooth *M*. *abscessus* suggest that intracellular processes are important. Transit to a degradative phagosome, or activation of autophagy are possible routes of intracellular elimination [41,42], without involvement of intracellular ROS. Resistance to ROS may be mediated in part by expression of catalase and superoxide dismutase enzymes whose genes have been identified in *M*. *abscessus* [43] (unpublished observation). In macrophage cultures, an anti-oxidant was observed to reduce *M*. *abscessus* growth, an effect attributed to enhanced phagosome-lysosome fusion [41]. In our hands, the anti-oxidant DPI did not affect survival of *M*. *abscessus*; this difference could be attributed to cell type-dependent processing, to the time-courses of infection, or different anti-oxidant targets. Killing of *M*. *tuberculosis* was reported to be insensitive to neutrophil oxidants [44]. Future experiments aim to determine the intracellular mechanism of killing of smooth *M*. *abscessus*.

Notably, mycobacteria can avoid intracellular processing [45]. Although not directly addressed in this study, subtle differences observed in ROS production, NET formation and phagocytosis might also reflect different abilities of neutrophils to detect and/or process the two morphotypes. While both morphotypes are similarly internalized, phagocytosis more profoundly influences killing of the smooth morphotype. These data are reminiscent of that seen in macrophages in which the smooth morphotype is eliminated while the rough morphotype survives intracellularly [13–15]. The different time periods of killing assays for neutrophils (hours) and macrophages (days) may be important, although extended incubation does not result in progressive clearance by neutrophils. In the case of neutrophils, sensitivity of the rough morphotype to extracellular killing mechanisms may overcome defects in intracellular killing. However, a short neutrophil life span suggests that intra-neutrophil survival may not constitute a major source of virulence in a neutrophil-rich environment. We have found that the smooth morphotype does not increase necrosis and has no effect on neutrophil apoptosis [33], suggesting that normal neutrophil clearance mechanisms may indirectly eliminate phagocytosed *M*. *abscessus*. However, the rough morphotype promoted necrotic cell death, suggesting a means for increased virulence during prolonged infection. Current studies are exploring neutrophil recognition and processing of *M*. *abscessus*.

Several limitations exist in this study. Assessing cfu of *M*. *abscessus* can be inconsistent due to unpredictable clumping of cultures and non-specific adherence to tubes. The culture conditions used in this study resulted largely in single cells and microcolonies based on microscopy (Figs 2 and 6), and initial dilutions were performed in detergent to minimize adherence. In extended experiments, *M*. *abscessus* were sonicated, instead of settled, to further reduce larger clumps. Interestingly, in settled cultures, phagocytosis played a bigger role in killing of rough *M*. *abscessus*, which suggest that physical properties of particles are important for neutrophil function [46,47]. Recent work indicates that in vivo the rough morphotype grows as cords and smooth *M*. *abscessus* grows as clumps [21], and not as individual cells, and in CF lungs colonization was observed in biofilm-like structures [48]; therefore, data obtained using single *M*. *abscessus* cell suspensions may poorly reflect natural infection conditions or the ability to predict host-response during disease. These differences emphasize the need for *in vivo* studies. Finally, while phagocytosis-dependent NET formation is a major mechanism for smooth *M*. *abscessus* clearance, we were unable to identify the exact mechanism of killing of rough *M*. *abscessus* by neutrophils. However, as almost no information is available on the interaction of *M*. *abscessus* and human neutrophils, and neutrophils are important for in vivo clearance in zebrafish [28], this study represents an essential advancement in the field.

These studies help define the development of *M*. *abscessus* innate immune responses and disease progression. In healthy individuals initial environmental smooth morphotype infections are detected and phagocytosed by resident macrophages, and subsequent neutrophil accumulation allows clearance of remaining *M*. *abscessus*. The limited clearance by neutrophils seen in our studies may be of little biological consequence if the inoculum is low and antimycobacterial activity is normal. However, bacterial may survive in immunocompromised individuals, or in cases where immune control could be physically limited, as in the case of bacteria protected in fomites or biofilms [4,33,48,49], a mode of growth that imparts immunoresistance and is confined mostly to the smooth morphotype [15]. Unknown signals promote conversion to the rough morphotype, which has virulent properties including immune activation, neutrophil necrosis, cell invasion, and macrophage intracellular survival [13–15,17,33]. In this case, the limited control of infection by neutrophils may lead to neutrophil death, extracellular bacterial growth, and continuing inflammation accompanied by extensive ROS release and other factors involved in tissue injury. Biofilms, aided by lysed neutrophils [33], and intracellular organisms can also act as reservoirs for continuing infection and immune activation. Excessive inflammation and bacterial burden may also overwhelm macrophage defense mechanisms. A cycle of mycobacterial growth and inflammation may therefore promote disease progression. The importance of neutrophils is highlighted by their prevalence at sites of *M*. *abscessus* infection. Studies of immune dysfunction on control of *M*. *abscessus* infection may provide insight into some of these possible mechanisms.

In summary, smooth and rough morphotypes of *M*. *abscessus* activate common neutrophil mechanisms of bacterial clearance, including ROS generation, NET formation, and phagocytosis. The known biochemical difference between morphotypes is expression of GPL on the cell wall. GPL-containing smooth *M*. *abscessus* is sensitive to intracellular and NET killing, while for GPL-deplete rough *M*. *abscessus* intracellular killing mechanisms are not required but NETs and an unidentified extracellular mycobactericidal factor(s) are important. Details of intracellular and extracellular killing mechanisms are the topic of future studies, which may help to define better strategies to clear *M*. *abscessus* infections.

## Supporting information

S1 Dataset Figure source data. (XLSX)

S1 Fig Cytokine release from *M*. *abscessus*-stimulated neutrophils. Neutrophils were exposed to smooth (Sm) and rough (R) *M*. *abscessus*, or left non-stimulated (NS) for the indicated times. Supernatants were isolated and (**A**) TNFɑ, (**B**) IL-1ß, (**C**) IL8, and (**D**) CCL4/MIP1ß measured by ELISA. The mean + SEM is represented from 9 independent experiments.
(PDF)
