# Supplementary material for: Neutrophil killing of Mycobacterium abscessus by intra- and extracellular mechanisms
Source: PLoS One. 2018 Apr 19;13(4):e0196120. doi: 10.1371/journal.pone.0196120 (PMC5909612; doi:10.1371/journal.pone.0196120)
Supplement: S1 Fig — Neutrophils were exposed to smooth (Sm) and rough (R) M. abscessus, or left non-stimulated (NS) for the indicated times. Supernatants were isolated and (A) TNFɑ, (B) IL-1ß, (C) IL8, and (D) CCL4/MIP1ß measured by ELISA. The mean + SEM is represented from 9 independent experiments. (PDF) [file pone.0196120.s002.pdf]

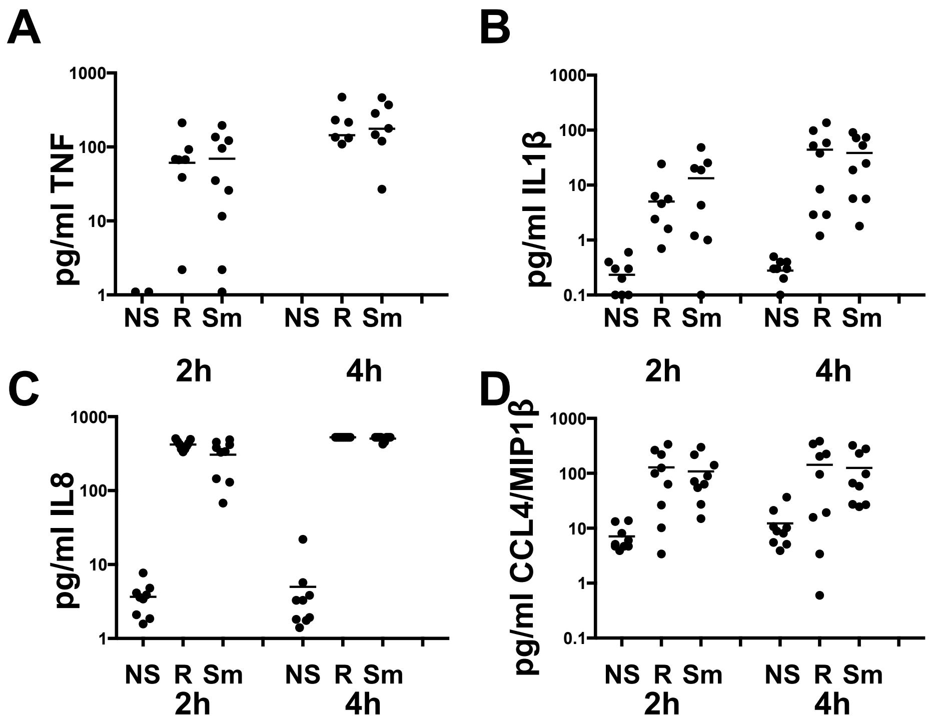

S1 Fig. Cytokine release from *M. abscessus*-stimulated neutrophils. Neutrophils were exposed to smooth (Sm) and rough (R) *M. abscessus*, or left non-stimulated (NS) for the indicated times. Supernatants were isolated and (**A**) TNFα, (**B**) IL-1ß, (**C**) IL8, and (**D**) CCL4/MIP1ß measured by ELISA. The mean ± SEM is represented from 9 independent experiments.
